# Anatomical relation between the accessory process and pedicle in the lumbar vertebrae

**DOI:** 10.1007/s12565-018-0432-3

**Published:** 2018-02-09

**Authors:** Ryutaro Shiboi, Shogo Hayashi, Shinichi Kawata, Zhong-Lian Li, Philipp Pieroh, Hisashi Koga, Yuichi Takano, Hirohiko Inanami, Masahiro Itoh

**Affiliations:** 1 Department of Anatomy, Tokyo Medical University, 6-1-1 Shinjuku, Shinjuku-ku, Tokyo, 160-8402 Japan; 2 Division of Orthopaedic Surgery, Oono Central Hospital, 3-20-3 Shimokaizuka, Ichikawa, Chiba 272-0821 Japan; 3 Department of Anatomy, School of Medicine, International University of Health and Welfare, 4-3 Kozunomori, Narita, Chiba 286-8686 Japan; 4 Department of Orthopedics, Trauma and Plastic Surgery, University of Leipzig, Liebigstrasse 20, 04103 Leipzig, Germany; 5 Department of Anatomy and Cell Biology, Martin Luther University of Halle-Wittenberg, Grosse Steinstrasse 52, 06097 Halle (Saale), Germany; 6 Iwai Orthopaedic Medical Hospital, 8-17-2 Minami koiwa, Edogawa-ku, Tokyo, 133-0056 Japan; 7 Inanami Spine and Joint Hospital, 3-17-5 Higashi shinagawa, Shinagawa-ku, Tokyo, 140-0002 Japan

**Keywords:** Accessory process, Computed tomography, Lumbar spine, Pedicle screw

## Abstract

The pedicle screw is one of the most common medical devices used in spinal surgery. Although there are well-established insertion points based on anatomical landmarks, such as the mammillary process and the transverse process, morphological data on the relationship between the accessory process and the pedicle are still scarce. To clarify this relationship, we recruited 50 cases of hernia of lumbar intervertebral disc, diagnosed using three-dimensional computed tomography of the lumbar vertebrae. We identified the pedicle isthmus in a transverse plane parallel to the upper endplate and measured the angles and distances from the tip of the accessory process to the intersection points at the medial or lateral surface, or at the midpoint between the two intersection points. In a sagittal plane showing the pedicle isthmus, we measured the wedging angle of the vertebral body as well as the angle from the tip of accessory process to the posterior edge of the upper endplate of vertebral body, or to the lower end of the pedicle root. We found that from the tip of the accessory process passing through the pedicle isthmus, a line should be directed 20 (± 6.6) degrees medially in the transverse plane and 5 (± 4.3) degrees cranially in the sagittal plane. This distance from the tip of the accessory process to the isthmus was 1.5 (± 0.3) cm. Our study provides a new anatomical basis for the use of the accessory process as a landmark for insertion of the pedicle screw.

## Introduction

Clinical success with pedicle screw systems was reported during the 1980s by many researchers (Cotrel et al. 1988; Louis 1986; Roy-Camille et al. 1986). The pedicle screw is one of the most common medical devices used for vertebral fusion and treatment of vertebral fracture (Bandela et al. 2013; Ikeuchi and Ikuta 2016; Su et al. 2017). Although three-dimensional (3D) navigation during surgery has significantly improved the accuracy of pedicle screw insertion (Luther et al. 2015), there are well-established insertion points based on anatomical landmarks, such as the mammillary/articular process, the transverse process, and the pars interarticularis (Oh et al. 2013). Nevertheless, during insertion of the pedicle screw there is a risk for violation of the zygapophyseal or facet joint, especially in the upper lumbar vertebrae, which could lead to adjacent segment degeneration (Laine et al. 1997). The risk may be increased in the presence of degenerative deformations of the spinal segments. For example, due to the changes in load transmission, a sagitalization of the superior articular process may result in a reduced distance from the facet joint to the mammillary process, thereby enhancing the risk for facet joint violation (Chung et al. 2007; Pal and Routal 1999; Zeng et al. 2015).

Various navigation systems have been developed in recent years to assist pedicle screw insertion, including C-arm-guided fluoroscopy and computed tomography (CT)-based navigation (Bandela et al. 2013; Su et al. 2017). Percutaneous pedicle screw placement with C-arm-guided fluoroscopy has enabled minimal invasiveness (Ikeuchi and Ikuta 2016). In terms of accuracy of the insertion, Ikeuchi and Ikuta (2016) reported that they found no statistically significant differences in insertion accuracy between the percutaneous pedicle screw placement and the conventional free-hand technique. The free-hand technique for placement of pedicle instrumentation, however, relies completely on the use of visible as well as palpable anatomic landmarks for its accuracy.

In Japan, orthopedic surgeons often use the accessory process as an anatomical landmark for pedicle screw insertion (Imae et al. 2010; Kasai et al. 2009; Suzuki and Shimizu 2009; Toyama et al. 2010). This landmark can be utilized in cases of severe degeneration and impairment of landmark identification to provide a more protective and safe insertion of the pedicle screw. Nonetheless, morphological data on the relationship between the accessory process and the pedicle are still scarce. Therefore, the objective of this study was to discern the detailed relationship between the accessory process and the pedicle isthmus in terms of their relative distances and angles, so as to provide a new anatomical basis for insertion of the pedicle screw.

## Materials and methods

Fifty patients (32 males, 18 females) diagnosed with a hernia of lumbar intervertebral disc were recruited to the study. Prior to enrollment, all patients willingly signed a form consenting to participation in the study and also acknowledged their awareness that there would be no disadvantage if they were to leave the study at any time. This study was approved by the Ethics Committee of Iwai Orthopedic Medical Hospital and was performed in accordance with the provisions of the Declaration of Helsinki 1995 (as revised in 2013).

The average age and height (± standard deviation [SD]) of the patients was 30.5 ± 6.7 (range 23–38) years and 167.6 ± 8.1 cm, respectively. The average body weight was 64.5 ± 13.1 kg. A wide range of CT scanning examinations were carried out before surgery, using the Light Speed Pro 16 CT Scanner (GE Healthcare, Chicago, IL, USA). The 3D reconstruction of the lumbar spine was achieved using OsiriX software (Rosset et al. 2004) with a slice thickness of 1.25 mm.

**Fig. 1:**
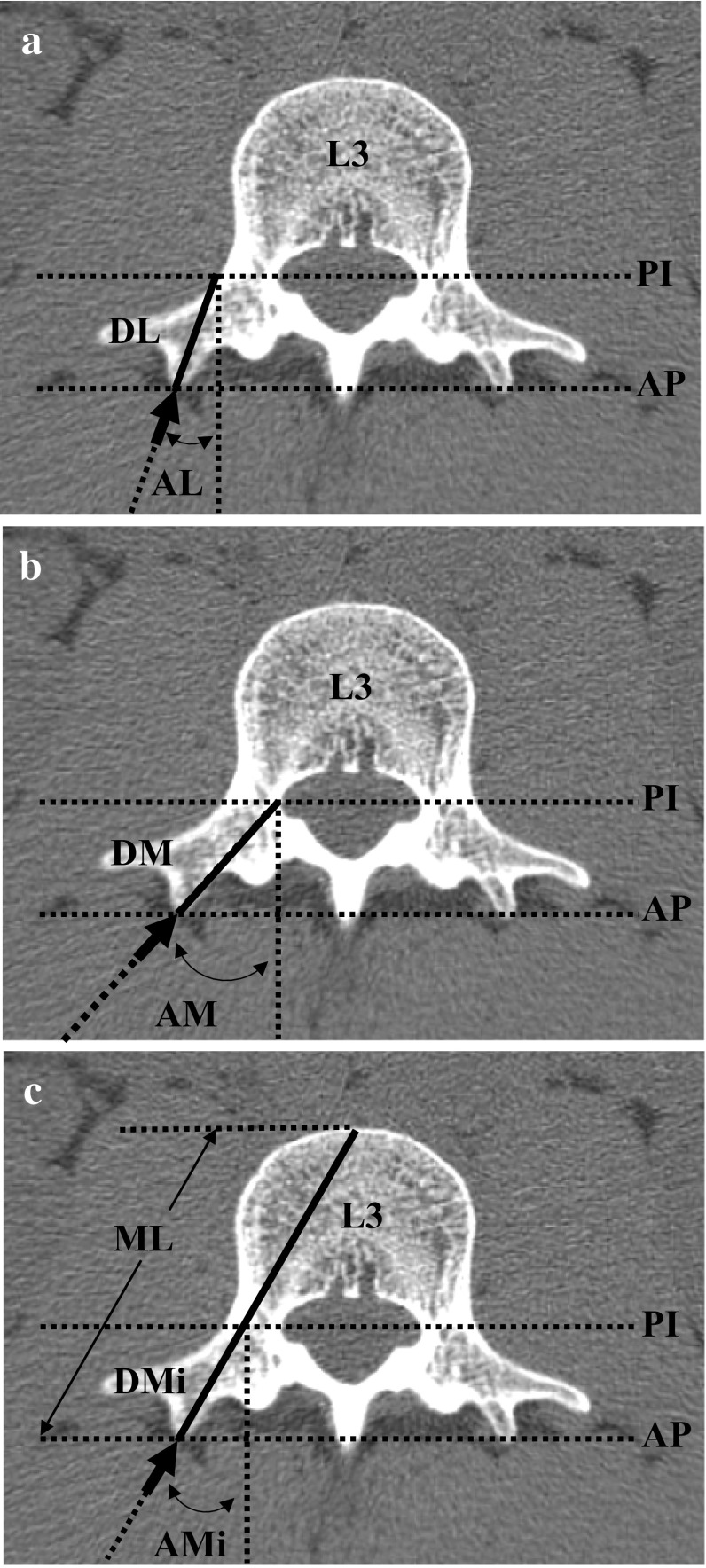
Measurement of angles and distances from the accessory process to the pedicle In a transverse plane of vertebra L3 showing the pedicle isthmus, the line connecting the accessory processes (*AP*) was drawn parallel to that passing the pedicle isthmus (*PI*), intersecting with the lateral and medial surface of the pedicle. Angles (*A*) and distances (*D*) were measured (solid lines) respectively from the tip of the accessory process (arrows): **a** to the intersection point at the lateral (*L*) surface of the pedicle (*AL* and *DL*), **b** to the intersection point at the medial surface (*M*) of the pedicle (*AM* and *DM*), and **c** to the midpoint between L and M (*AMi* and *DMi*). Maximum length (*ML*) represents the extension line of DMi to the surface of the vertebral body

The measurement was designed to identify trajectory parameters for a line from the tip of the accessory process passing through the centroid of the pedicle isthmus. In a transverse plane that was parallel to the upper endplate of the vertebral body and showing the pedicle isthmus (Fig. 1a), the tips of the accessory process on both sides were determined using multiplanar reconstruction, where they protruded most in the dorsal direction. Parallel to the straight line connecting the tips of the accessory process on both sides, another straight line was drawn that intersected with the outermost medial (M) and lateral (L) surfaces of the pedicle (Fig. 1a–c). The angles (A) and distances (D) were measured from the tip of the accessory process to the intersection point at the lateral (AL and DL, respectively) or medial surface (AM and DM, respectively) of the pedicle, or the midpoint (AMi and DMi, respectively) between the medial and lateral intersection points. Additionally, the extension of line of DMi (Fig. 1b) would intersect with the surface of the vertebral body; between this intersection point and the tip of the accessory process, the distance was defined as the maximum length (ML).

**Fig. 2:**
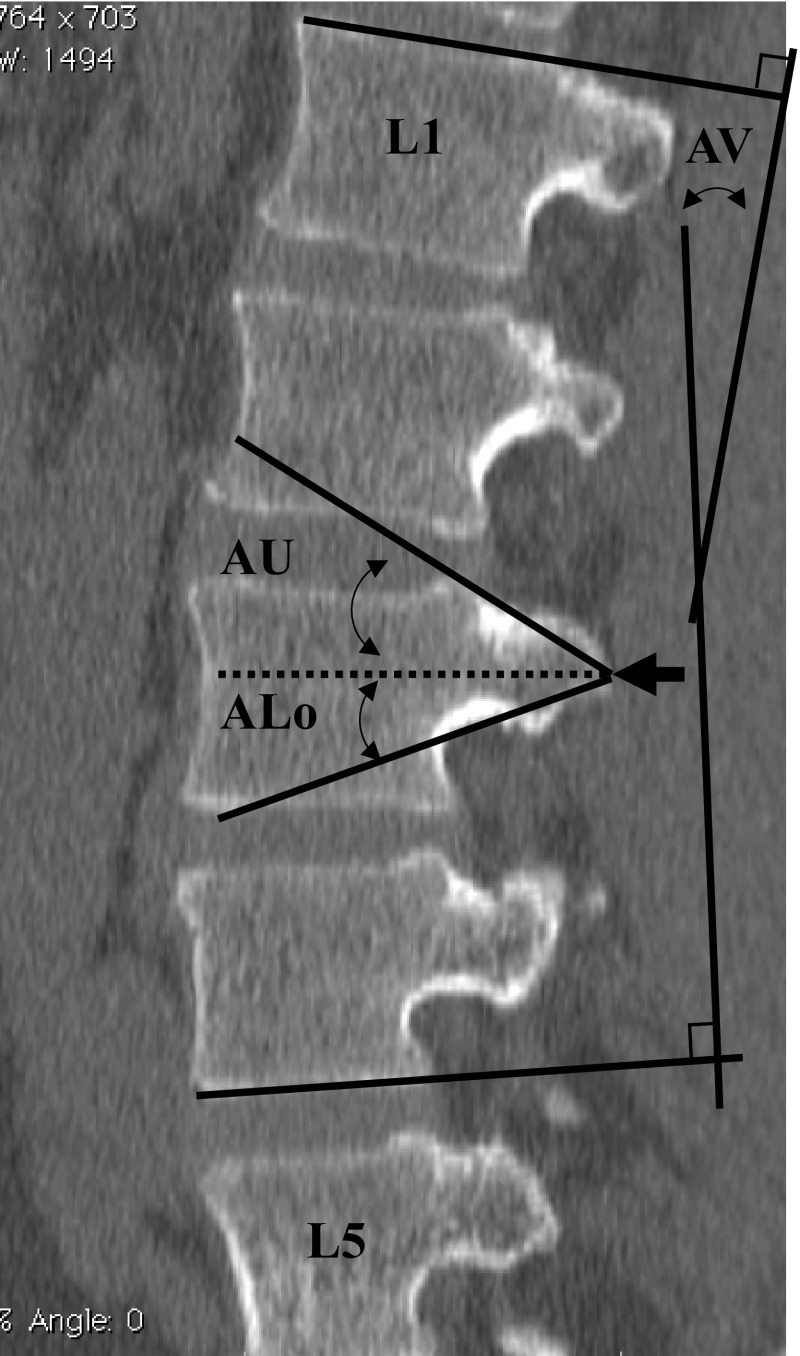
Measurement of angles and distances from the accessory process to the pedicle. From the tip of the accessory process (arrow) and parallel to the upper endplate (dashed line), three angles were measured in a sagittal plane of the lumbar (L1–L5) vertebrae showing the pedicle isthmus: wedging angle of the vertebral body (*AV*), angle to the posterior edge of the upper endplate of the vertebral body (*AU*), and angle to the lower end of the pedicle root (*ALo*)

On the other hand, in the sagittal plane (Fig. 2) showing the pedicle isthmus, the angle (A) was measured from the tip of the accessory process to the posterior edge of the upper (U) endplate of the vertebral body (AU), or to the lower end (Lo) of the pedicle root (ALo). The wedging angle of the vertebral body (AV) was measured from the upper endplate of L1 through the lower endplate of L2–L5 using the Cobb method (Cobb 1948). All measurements of angles and distances were carried out on both the right and left sides.

All values were presented as the average ± SD whenever applicable. All data was analyzed using one-way analysis of variance. Tukey–Kramer multiple comparison tests were used for the data with significant difference. A *P* value ≤ 0.05 was considered to be statistically significant.

## Results

**Fig. 3:**
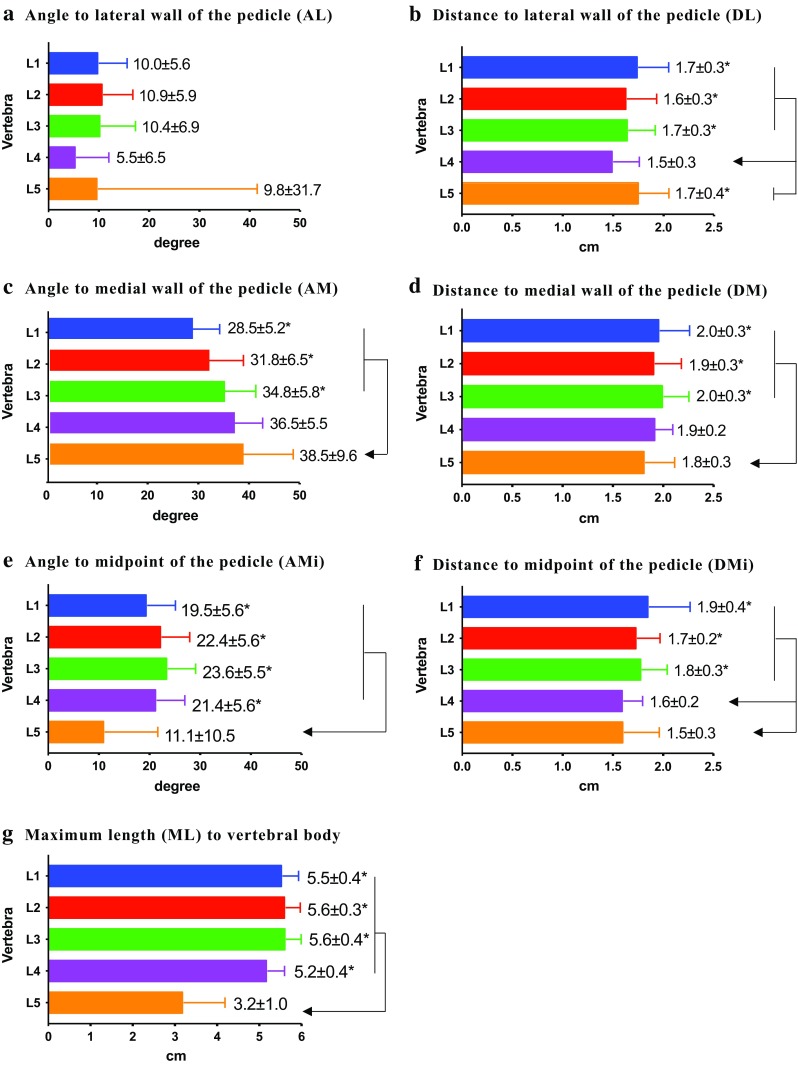
In a transverse plane showing the pedicle isthmus, angles (*A*) and distances (*D*) from the tip of the accessory process were measured.** a**,** c**,** e** The three angles measured were those to the lateral surface of the pedicle (*AL*) (**a**), those to the medial surface of the pedicle (*AM*) (**c**), and those to the midpoint between AL and AM (*AMI*) (**e)**.** b**,** d**,** f **The distances measured were those to the lateral surface of the pedicle (*DL*) (**b**), those to the medial surface (*DM*) of the pedicle (**d**), and those to the midpoint between DL and DM (*DMi*) (**f**).** g** Maximum length (*ML*). The values are presented as the average ± standard deviation (SD).* Asterisk* indicates a significant difference (*P* ≤ 0.05) among specified vertebrae

**Fig. 4:**
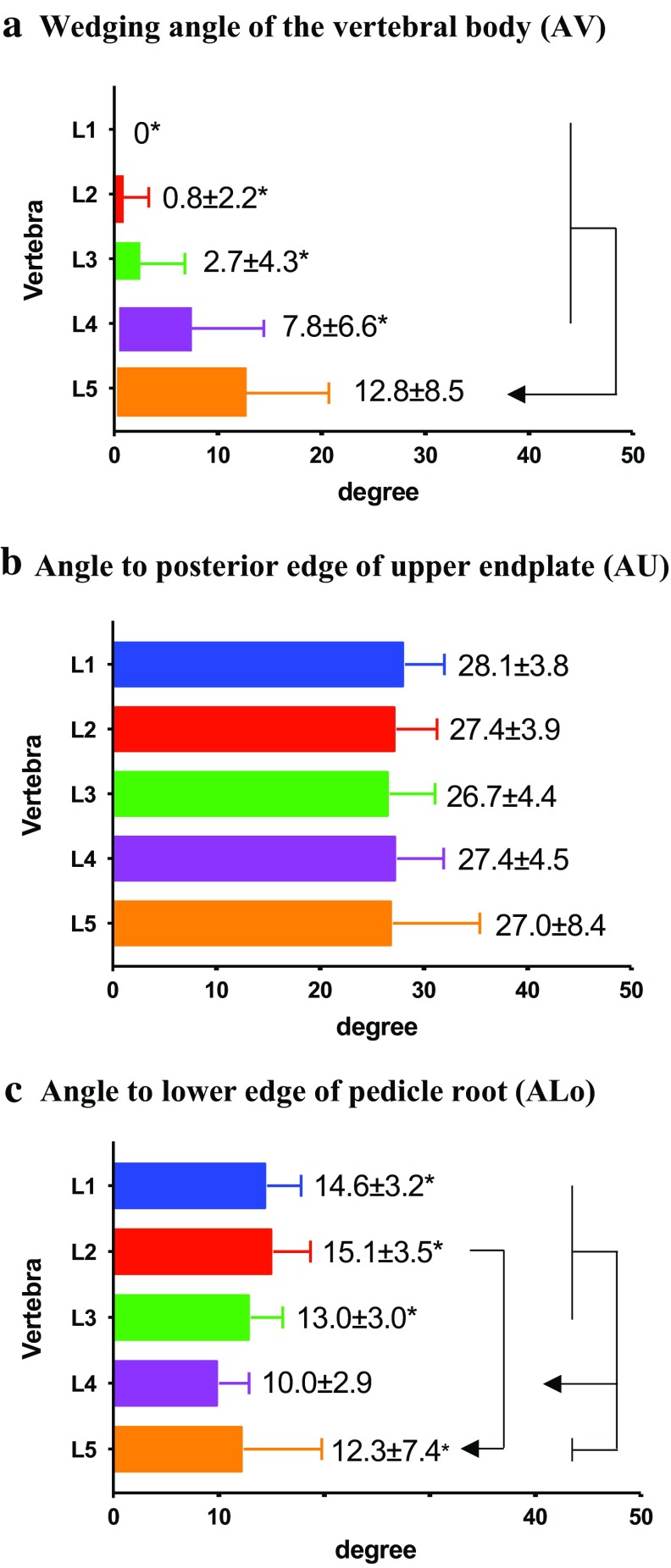
Morphological characteristics of vertebrae L1 (**a**) and L5 (**b**) in a transverse plane parallel to the upper endplate. The tip of the accessory process (arrowheads) and the pedicle isthmus were observed. Arrows indicate difference in the shape of vertebral body. Note that from the tip of the accessory process to the pedicle or to the surface of vertebral body (dashed lines), the maximum length (*ML*) was different

Because no significant side (left vs. right) difference was detected in all values (data not shown), we considered the right and left measurements to be independent values for the statistical analyses. From the tip of the accessory process, three types of angles (A) were measured in the transverse plane. To the lateral (L) surface of the pedicle isthmus (Fig. 1a), the first angle AL (Fig. 3a) was 9.3 (± 5.2) degrees on average, with no significant difference among lumbar vertebrae. To the medial (M) surface of the pedicle isthmus, the next angle AM (Fig. 3c) increased from 28.5 (± 5.2) degrees in L1 to 38.5 (± 9.6) degrees in L5, which is significantly larger than that for L1 to L3 but not for L1 to L4. The third angle AMi (Fig. 3e) was defined for the line passing approximately through the midpoint (Mi) of the pedicle isthmus. Among L1–L4, the AMi angle varied between 19.5 (± 5.6) and 23.6 (± 5.5) degrees without any significant difference. All of these angles, however, were significantly larger than that in L5 (11.1 ± 10.5 degrees). This difference is due to the gradual increase in the diameter of the pedicle in the direction of the sacrum (Fig. 4), so that the lines passing through the midpoint would shift laterally. From the tip of the accessory process, therefore, a line should be directed 20 (± 6.6) degrees medially in the transverse plane so as to pass the pedicle isthmus.

In the same transverse plane, three distances were measured from the tip of the accessory process to the pedicle isthmus. To the lateral (L) surface of the pedicle, the first distance DL (Fig. 3b) was 1.5 (± 0.3) cm in L4, which is significantly shorter than that in L1–L3 and L5, which varied between 1.6 (± 0.3) and 1.7 (± 0.4) cm without any significant difference among them. To the medial (M) surface of the pedicle, the next distance DM (Fig. 3d) varied between 1.9 (± 0.3) and 2.0 (± 0.3) cm in L1–L3, with no significant difference, and between 1.8 (± 0.3) and 1.9 (± 0.3) cm in L4–L5, also with no significant difference. It should be noted that DM in L5 was 1.8 (± 0.3) cm, which is significantly shorter than that in L1–L3. The third distance measure was DMi (Fig. 3f), a line approximately connecting the midpoint (Mi) of the pedicle isthmus. Among L1–L3, the distances varied between 1.7 (± 0.2) and 1.9 (± 0.4) cm, which is significantly longer than that in L4–L5, which varied between 1.6 (± 0.2) and 1.5 (± 0.3) cm. Additionally, the ML (Fig. 3g) parameter was designed to keep the extension line of DMi within the vertebral body. The length in L5 was 3.2 (± 1.0) cm, which is significantly shorter than 5.5 (± 0.4) cm in L1–L4, which varied between 5.2 (± 0.4) and 5.6 (± 0.4) cm. The variations in the distances may be related to the morphological transition in the lumbar vertebrae (Fig. 4). In general, from the tip of the accessory process, a line of 1.5 (± 0.3) cm in length on average would remain within the pedicle isthmus.

**Fig. 5:**
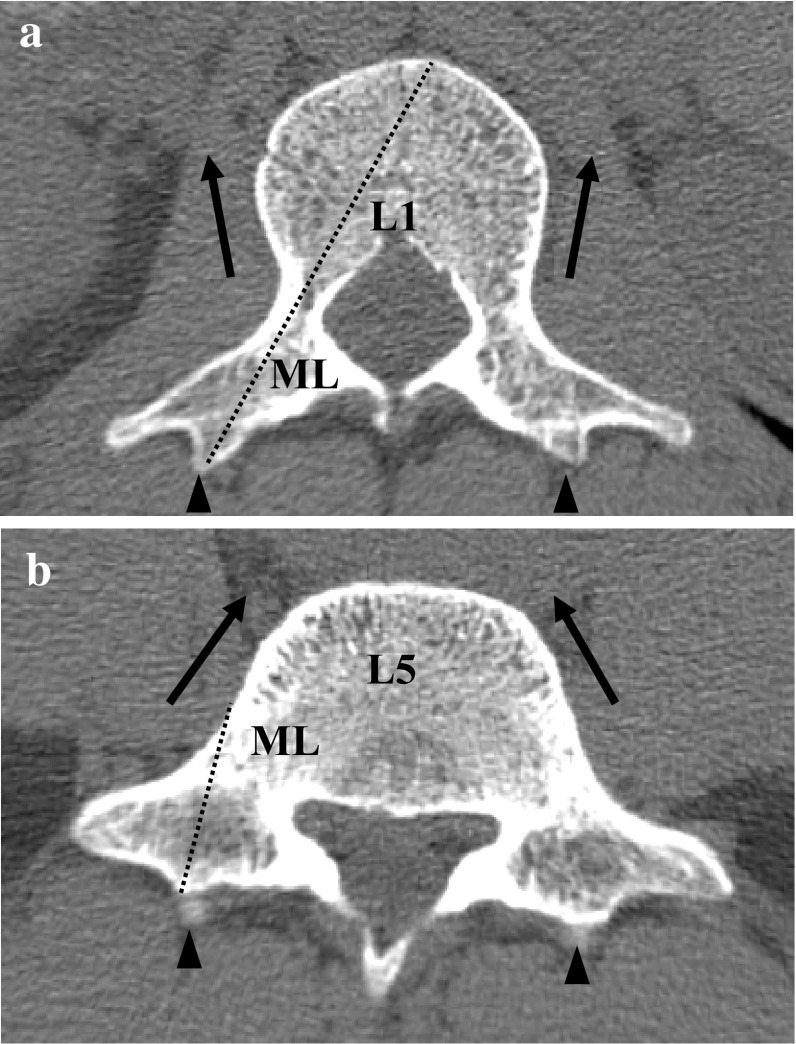
From the tip of the accessory process and parallel to the upper endplate three angles were measured in a sagittal plane showing the pedicle isthmus. **a** The wedging angle of the vertebral body (AV), **b** the angle to the posterior edge of the upper endplate of vertebral body (AU), **c** the angle to the lower edge of the pedicle root (ALo). The values are presented as the average ± SD.* Asterisk* indicates a significant difference (*P* ≤ 0.05) among specified vertebrae

On the other hand, three angles were measured in the sagittal plane showing the pedicle isthmus. The first one is the wedging angle (A) of the vertebral body AV (Fig. 5a), which increased from 0 degrees in L1 to 7.8 (± 6.6) degrees in L4, and finally to 12.8 (± 8.5) degrees in L5, with the latter measurement significantly larger than all the others. The second angle is for the line pointing to the posterior upper edge of the vertebral body, i.e., the angle AU (Fig. 5b); it was kept between 26.74 (± 4.4) and 28.1 (± 3.1) degrees in all vertebrae without significant difference. To the lower edge of the pedicle root, the third angle, i.e., ALo (Fig. 5c), varied between 13.0 (± 3.0) and 15.1 (± 3.5) degrees among L1–L3 and L5 and was significantly larger than that in L4 (10.0 ± 2.9 degrees). In addition, the largest ALo was identified in L2 (15.1 ± 3.5 degrees), the only angle even significantly larger than that in L5. Based on these angles, a line should point 5 (± 4.3) degrees cranially in order to pass the pedicle isthmus.

## Discussion

The aim of our study was to analyze the detailed relationship between the accessory process and the pedicle isthmus in terms of their relative angles and distances. We found that from the tip of the accessory process, a line should be directed 20 (± 6.6) degrees medially in a transverse plane parallel to the upper endplate, and 5 (± 4.3) degrees cranially in the sagittal plane, so as to pass the pedicle isthmus. The length of the line from the tip of the accessory process to the isthmus is 1.5 (± 0.3) cm. This line would not penetrate the L1–L4 vertebral body so long as it was kept at 5.5 (± 0.4) cm in length.

The results from our study are in excellent agreement with the clinical definition for insertion of the pedicle screw in Japan. Toyama et al. (2010) suggested that the entry point should be located 1 mm medially or laterally from the accessory process and 15–20 degrees medially for the lower lumbar vertebrae, while keeping parallel to the disc in the sagittal plane. Imae et al. (2010) proposed that the entry point should be defined medially/superiorly to the tip of the accessory process and that it should be inserted 5–15 degrees (10–20 degrees for L5 only) medially in the transverse plane. Suzuki and Shimizu (2009) reported that the entry point should be on the top edge of the accessory process, and Kasai et al. (2009) proposed that the entry point should be at the apex of the bony triangle formed by the medial articular process of the accessory process. Although it is a common clinical practice to use the accessory process in localization of the pedicle entry point, especially in Japan, there is no comprehensive description of the relationship between the accessory process and the pedicle. However, based on experiences in clinical practice and the results of our study, the accessory process could become an even more practical landmark for identifying the entrance point for lumbar pedicle screw.

The free-hand technique for accurate placement of the pedicle instrumentation relies completely on both the visible and palpable anatomic landmarks. One of the major entrance points is located at the intersection between the lateral border of the mammillary/superior facet joint and the midline of the transverse process (Magerl 1984; Roy-Camille et al. 1986; Weinstein et al. 1988). It is worth noting that the major difference is that the accessory processes are situated lateral and inferior to the mammillary process. The mammillary process, including the articular facet, is large and indeed easily palpable. Nevertheless, in the presence of degenerative deformations of the spinal segments, a sagitalization of the superior articular process may result in a reduced distance from facet to the mammillary process and thereby enhance the risk for facet joint violation (Pal and Routal 1999). Matsukawa et al. (2016) reported that there is significant difference in the incidence of cranial facet joint violation between the age of 57.9 ± 17.7 and 71.7 ± 11.3 years and that this difference is significantly affected by pre-existing facet joint degeneration. Freehand pedicle screw insertion usually depends on several bony landmarks to localize an entry point, and when the accessory process, lying lateral-inferior to the facet is utilized, it tends to decrease the incidence of complications (Kasai et al. 2009).

A limitation of our study is that the cases (images) analyzed were those from patients younger than 40 years; therefore, the structural changes that may occur in elderly people are not known. Furthermore, this investigation used CT data from patients in the supine position. In practice, pedicle screw surgery is performed with subjects lying in prone position; therefore, the value of the sagittal angle in particular is predicted to be different from that during actual pedicle screw surgery.

## Conclusion

We have elucidated a detailed relationship between the accessory process and the lumbar pedicle. An anatomical basis was established for the accessory process to be used as a landmark for pedicle screw insertion. The landmark would be effective in reducing the risk of zygapophyseal joint violation during the pedicle insertion.
